# The possible effects of chili peppers on ADHD in relation to the gut microbiota

**DOI:** 10.3389/fnut.2025.1551650

**Published:** 2025-02-04

**Authors:** Yinyue Li, Jing Feng, GuangYao Ding, Lin Deng, Ying He, Qiongqiong Zhang, Jianhui Wang, Xia Chen

**Affiliations:** Department of Pediatrics, Child and Adolescent Psychiatric Center of Jiangbei Campus, The First Affiliated Hospital of Army Medical University (Army 958th Hospital), Chongqing, China

**Keywords:** ADHD, gut microbiota, diet, MGBA, chili peppers

## Abstract

Attention deficit hyperactivity disorder (ADHD) is a common neurodevelopmental disorder, which is characterized by inattention, impulsivity and hyperactivity. Although the etiology and pathogenesis of ADHD are not fully understood, existing studies have shown that it may be related to genetic factors, environmental factors, abnormal brain development, and psychosocial factors. In recent years, with the concept of microbioa-gut-brain axis (MGBA), more and more studies have begun to pay attention to the effect of gut microbiota on ADHD. Dietary structure can significantly change the diversity and abundance of gut microbiota. Therefore, dietary supplements or food additives to regulate gut microbiota have become one of the potential ways to treat ADHD. Peppers, as an important dietary component, have potential value in regulating gut microbiota. Among them, capsaicin (8-methyl N-vanillyl-6-noneamide, CAP), as a key active component of peppers, has been shown to have potential therapeutic effects on central nervous system (CNS) diseases such as Parkinson’s disease, epilepsy, and depression. In addition, much attention has been paid to the beneficial effects of CAP on gut microbiota. Chili peppers contain not only CAP, but also rich in vitamin C and fatty acids, all of which may ameliorate ADHD by modulating the gut microbiota. This finding not only provides a potential treatment for ADHD, but also provides a new perspective to expand the research and clinical treatment of ADHD pathogenesis. Although current research on the potential therapeutic effects of chili peppers on ADHD is still at an early stage and requires further verification through larger-scale and more rigorous controlled studies, its potential clinical value cannot be ignored.

## 1 Introduction

Attention deficit hyperactivity disorder (ADHD) is one of the most common neurodevelopmental disorders in children and adolescents, which is characterized by excessive activity, attention deficit and impulsive behavior. ADHD seriously affects patients’ academic performance, interpersonal relationships, and reduces their quality of life. Studies show that about 30–50% of children continue to show symptoms into adulthood (1). The etiology of ADHD is multifactorial and includes genetic predisposition, environmental influences, abnormal brain development, and psychosocial factors. Currently, methylphenidate and atomoxetine are the most commonly used drugs, but these drugs are often accompanied by adverse reactions, such as nausea, vomiting, loss of appetite, mood instability and sleep difficulty. In recent years, a large number of studies have shown a bidirectional interaction between the gut and the brain, a mechanism known as the microbiome-gut-brain axis (MGBA) (2). The gut microbiota is a key player in the gut-brain axis (2), and its composition and function can influence the onset and progression of ADHD (3). Diet is one of the important factors shaping the composition and metabolic activity of the gut microbiota (4). Therefore, interventions through diet may have a positive impact on the management of ADHD. Researchers have begun to pay attention to the potential role of dietary intervention and gut microbiota in the treatment of ADHD.

Studies have demonstrated the association between diet and ADHD, and a balanced diet or nutritional supplements may be a potential method to control ADHD. As a common dietary ingredient, chili peppers are rich in vitamin C, capsaicin (8-methyl N-vanillyl-6-noneamide, CAP), fatty acids, and other active ingredients, which have anti-inflammatory, anti-oxidative, anti-cancer, and adjusting gut microbiota (5–9). Recent studies have shown that CAP has beneficial effects on central nervous system (CNS) diseases such as Parkinson’s disease, epilepsy, stroke, and depression by reducing oxidative stress and improving neuroinflammation (10–14). Vitamin C has neuroprotective effects and also serves as an adjunct in the redox chain reaction required for neurotransmitter synthesis. This has proven beneficial for brain diseases such as stroke and epilepsy (15). In addition, disorders of fatty acid metabolism are closely related to the pathogenesis of neuropsychiatric disorders (16). Next, this study will explore the relationship between diet, gut microbiota, and ADHD, and analyze the effects of peppers and their components on ADHD through the gut microbiota and brain-gut axis. This provides a theoretical basis for further human clinical studies to verify the potential benefits of long-term consumption of peppers on ADHD.

## 2 Diet can affect the gut microbiota

Gut microbiota refers to the entire microbial ecosystem that inhabits the gastrointestinal tract of mammals, including bacteria, fungi, viruses, and protozoa (17). This complex and dynamic system plays a critical role in human health and disease. The initial colonization of microbiota begins at birth, and the mode of delivery, feeding pattern, dietary structure, and environmental factors all have significant effects on the formation of early gut microbiota (18). During the early stages of life, the gut microbiota rapidly diversifies and plays a key role in the development of the immune system (19, 20). Into adulthood, although the microbiota remains generally stable, its composition and function can still be affected by external factors, among which diet is one of the most important factors (21, 22).

Diet can alter the composition and diversity of the gut microbiota through MGBA, which in turn affects the interactions between the brain, gut, and microbiota, which has important implications for brain health and a variety of neurological diseases (23). Healthy diet (rich in dietary fiber and multiple sources of viable bacteria) is capable of promoting not only an increase in gut microbial diversity but also the production of short-chain fatty acids (SCFAs) and other beneficial bioactive compounds that contribute to gastrointestinal health, metabolic health, and brain function (24, 25). In contrast, western diet dominated by processed foods leads to unhealthy changes in the gut microbiota and is associated with disorders such as psychiatric disorders, gastrointestinal disorders, metabolic disorders (26), and obesity (27). A prospective randomized controlled trial in healthy individuals showed that diet rich in fermented foods was able to enhance the diversity of the microbiota and significantly reduce the levels of proinflammatory cytokines such as serum interleukin (IL-6) (28). High-fiber diet significantly alters the composition of the gut microbiota, leading to a large increase in the number of beneficial bacteria such as *Lactobacillus* and *Bifidobacterium* (29). Two studies have established an association between the Mediterranean diet and specific microbial communities, finding increased populations of beneficial bacteria such as *Faecalibacterium prausnitzii* and *Roseburia* spp. It also reduces the abundance of *Collinsella aerofaciens*, and *Ruminococcus* (30, 31). Diet-induced changes in the gut microbiota are associated with increased SCFAs and decreased metabolic by-products such as ethanol and carbon dioxide. The influence of diet is present throughout the life span, and modulation of MGBA is a promising strategy for the prevention and treatment of mental health disorders.

## 3 The association between the gut microbiota and ADHD

Numerous studies have demonstrated that gut microbiota plays a crucial role in diseases like ADHD, Alzheimer’s disease (AD), autism, epilepsy, etc. (3, 32, 33). The gut microbiota can regulate the levels of neurotransmitters, affect the function of the hypothalamic–pituitary–adrenal (HPA) axis, and alter the permeability of the blood–brain barrier. These mechanisms constitute the key links of the brain-gut axis in the pathophysiological process of ADHD. The gut microbiota of ADHD patients was transferred to mice by researchers and found that the structural integrity of white matter and gray matter regions (such as the internal capsule and hippocampus) in the mice was lower, and there was a significant correlation between the structural integrity of white matter and the differentially expressed microbial community (34). Tengeler et al. (34) demonstrated that the transfer of gut microbiota from individuals diagnosed with ADHD to mice could induce behavioral modifications, which were not evident when utilizing gut microbiota from individuals without ADHD. Individuals with ADHD and healthy controls have different gut microbiota compositions (35, 36). The *Lactobacillus* family has been shown to be consistently lower in those with ADHD than in their healthy counterparts (3). Prehn-Kristensen et al. (37) have proved that the α diversity of the gut microbiota in adolescent ADHD patients was lower, and the hyperactivity symptom score was negatively correlated with α diversity. Researchers (38) conducted a study on the gut microbiota while sequencing 16S rRNA genes from the V4 region, and demonstrated that children and adolescents diagnosed with ADHD have different gut microbiota signatures from unrelated controls.

## 4 Beneficial effects of diet on the gut microbiota in ADHD

Dietary therapy has significant positive effects on a variety of neurological disorders, including depression, Parkinson’s disease, autism, ADHD, and epilepsy (39–42). The Mediterranean diet has long been regarded as a positive contributor to health. Studies have shown that intervention with a Mediterranean diet in children with ADHD significantly improves impulsiveness and hyperactivity in 8 weeks (43). Liu et al. (44) used mice as an animal model to study the effect of ketogenic diet (KD) on ADHD, and found that compared with the normal diet group, the KD group had a significant increase in the abundance of *Ruminococcus_gauvreauii_group*, *Bacteroides*, *Bifidobacterium*, and *Blautia*. However, the abundance of *Lactobacillus*, *Romboutsia*, *Facklamia*, and *Turicibacter* was significantly reduced in the KD group. According to research, KD can help alleviate behavioral disorders associated with ADHD by modulating the gut microbiota. One study evaluated the effect of the elimination diet on ADHD symptoms in children and showed that the elimination diet resulted in significant improvements in ADHD symptoms and the effect lasted longer (45). In addition, dietary deficiency of essential nutrients affects changes in synapse number and myelin integrity, thereby affecting neurodevelopmental processes, including the formation of hippocampal learning and memory pathways and the maturation of executive functions, ultimately leading to negative consequences associated with ADHD (46). A systematic review of randomized clinical trials has shown that iron and zinc deficiencies are associated with the onset of ADHD, which can be ameliorated by dietary interventions (47). These studies provide evidence that ADHD can be improved by dietary interventions, which opens up the possibility of future clinical treatment of ADHD. The microbiome and its metabolites are influenced by changes in diet and host physiological function. Therefore, different dietary structures may contribute to changes in children with ADHD by regulating the gut microbiota. The microbiome and its metabolites are influenced by changes in diet and host physiological function. Therefore, different dietary structures may contribute to changes in children with ADHD by adjusting the gut microbiota.

## 5 Chili peppers may be beneficial for ADHD

As a widely used vegetable and spice, peppers are rich in bioactive compounds, such as CAP, vitamin C, and fatty acids (24, 25). Since the 20th century, the production and consumption of chili peppers have gradually increased worldwide, making them an indispensable ingredient in numerous dishes (48, 49). Recent studies have shown that the active components in chili peppers exhibit a variety of biological properties *in vitro* and *in vivo* experiments, such as lipid-reducing, antioxidant, and anti-inflammatory effects (50–52). However, current research on the relationship between peppers and ADHD is still in its preliminary stage. Our study aimed to investigate the effects of chili peppers and its main components on ADHD. The prevalence of ADHD in China is lower than the global average, which may be related to dietary habits, cultural differences and economic development level. In China, especially Yunnan, Guizhou and Sichuan, the proportion of people who like spicy food is high, and the trend is steadily growing. Major components in chili peppers, such as CAP, vitamin C, and unsaturated fatty acids, have been shown to have the potential to modulate the gut microbiota. Based on the MGBA theory, a bidirectional interaction between the gut and the brain, we considered that chili peppers might also improve ADHD symptoms by modulating the gut microbiota.

### 5.1 CAP may ameliorate ADHD through the brain-gut axis

CAP is a vanillic acid derivative found mainly in the genus Capsicum and is a key contributor to its pungent taste. The mechanism of CAP activity covers both receptor-dependent and non-receptor-dependent pathways, but its protective effect against neurodegeneration is mainly through transient receptor potential vanilloid 1 (TRPV1). In the human CNS, TRPV1 is mainly found in the cerebral cortex, striatum and hippocampus. CAP can cross the blood–brain barrier and directly act on the nervous system via TRPV1 (53), participating in the neural pathways of the brain-gut axis. CAP also affects brain neurons and glial cells through TRPV1-dependent and independent mechanisms and plays a role in regulating autophagy, synaptic transmission and plasticity, and cognitive function (54, 55). CAP primarily reduces oxidative stress, alleviates pain and cognitive impairment, protects against cardiovascular disease, stroke, obesity, hypertension, and cancer via TRPV1 (56, 57). In addition, intestines are richly innervated by afferent sensory nerves expressing TRPV1 channels, and their activation plays an important role in the gut function regulation (58–60). CAP can stimulate TRPV1 receptors and induces local release of neuropeptides such as Substance P or gene-related peptide (58–61). These neuropeptides may regulate the composition and structure of the gut microbiota by altering the inflammatory and immune environment of the gut (62).

There has been an increasing amount of evidence suggesting that CAP can affect the composition, abundance, and function of the gut microbiota. It reduced hippocampal cell damage and improved brain tissue metabolism by enhancing the function of the gut-brain axis (63). Studies have shown that CAP increases the ratio of *Firmicutes/Bacteroidetes* bacteria and the number of *Clostridium* bacteria, which leads to an increase in peripheral 5-hydroxytryptamine (5-HT; serotonin) levels through microbial transfer (64). Mahalak et al. (5) found that adding CAP to an *in vitro* culture model of the human gut microbiota increased microbial diversity and increased the abundance of certain SCFAs, particularly butyric acid. But the study was conducted *in vitro* and did not consider the influence of host functions on the study results. In two separate *in vivo* studies conducted on mice, the inclusion of dietary CAP led to a decrease in weight gain and food consumption. Additionally, it enhanced the abundance of important intestinal microbial genera like *Bacteroidetes*, *Akkermansia*, and *Prevotella*, while simultaneously lowering the levels of *Escherichia* and *Salmonella genera* (65, 66). Ryu et al. (63) explored how Korean chili paste (kochujang) containing an appropriate amount of CAP could alleviate memory impairment in rats through the enteric-brain axis, and they found that compared to the control group, kochujang (Medium-CPS) intake decreased *Clostridium* and *Solibaculum* and increased *Blautia* and *Lachnoclostridium* belonging to the family *Lachinospraceae* compared to the Control group. These changes affected the metagenome function related to brain function and inflammation. The consumption of red peppers in kochujang has been shown to improve inflammation in rats with dextran sulfate sodium-induced colitis, modulating gut microbiota by decreasing harmful *Enterococcus faecalis* and *Staphylococcus sciuri* (67). An animal study has shown that CAP can alleviate Lipopolysaccharid (LPS) -induced depressive-like behavior and reduce TNF-α levels by enhancing the relative abundance of certain gut microbiota, such as *Ruminococcus* and *Prevotella* (14). CAP can increase beneficial bacteria such as *Firmicutes* and reduce harmful bacteria such as *Enterococcus faecalis* and *Staphylococcus sciuri* by changing the composition and abundance of intestinal flora. This can increase neurotransmitters (5-HT, DA), metabolites (SCFAs), etc. This affects the various pathways of the brain-gut axis and acts as an anti-inflammatory and neuroprotective agent to improve ADHD. But more molecular studies are needed to elucidate the underlying subtle mechanisms of gut-capsaicin interactions.

CAP has been shown to have potential neuroprotective properties, including improving cognitive function and preventing neurodegenerative diseases (67–70). The beneficial effects of CAP in Parkinson’s disease and depression have been described (71). CAP is also capable of enhancing the neural function of stroke patients (71). A study of the diet and cognitive status of 338 middle-aged and older adults shows that long-term intake of CAP can effectively improve cognitive performance in AD patients. This suggests that CAP could be a potential drug option for prevention and treatment of AD (72). In a crossover randomized controlled trial, patients with amyotrophic lateral sclerosis (ALS) experienced significant relief of ALS-related dysphagia after the administration of CAP (73). These researches suggest that CAP, as a common food additive, may have potential benefits for neurological diseases. It is currently believed that an imbalance in the gut microbiota may be associated with the onset of ADHD (35, 36). We believe CAP may be an emerging potential treatment for ADHD patients.

### 5.2 Beneficial effects of vitamin C on gut microbiota

The initial response of many individuals to chili peppers is that they are hot, but beyond this characteristic, they also hold a remarkable title - they are the top source of vitamin C among vegetables. The amount of vitamin C in different types of chili peppers varies. The amount of vitamin C found in different peppers genotypes varies between 43 and 247 mg for every 100 g of fresh fruit (74). The highest is sweet peppers, whose vitamin C content is four times that of oranges and 43 times that of apples. Vitamin C is a very important antioxidant and free radical scavenger that can promote iron ion absorption (75), participate in collagen synthesis, hormone synthesis, and carnitine synthesis (76). Additionally, it plays an important role in the function and regulation of the immune system (77). The health benefits of vitamin C have been widely proven, and the strong correlation between the gut microbiota and host health or various diseases has been widely reported, so exploring the known regulation of vitamin C on the gut microbiota is crucial.

Research indicates that after a two-week regimen of high-dose vitamin C supplementation, there is a notable rise in the relative abundance of the bacterial groups *Lachnospiraceae* (other) among healthy individuals. In contrast, both the Bacteroidetes phylum and Enterococcus genus exhibit a significant reduction (78). The bacterial groups *Lachnospiraceae* belongs to the Firmicutes phylum, and is a major bacterial group in the gut microbiota of healthy human subjects (79, 80). *Lachnospiraceae* is one of the main producers of SCFAs. These are known bacterial metabolites with multiple anti-inflammatory and antioxidant effects (81). Another study used a mouse model of rheumatoid arthritis to validate the regulatory role of vitamin C in modulating gut microbiota balance, which improved the alpha and beta diversity of the bacterial community, especially increasing the abundance of the genus *Lachnospira* and increasing the production of SCFAs (82). Vitamin C reconfigures the gut microbiota, improves the intestinal environment, reduces inflammatory responses and oxidative stress in the paraventricular nucleus of the hypothalamus, thereby weakening sympathetic nervous activity in hypertensive rats and lowering blood pressure (83).

Vitamin C increases the abundance of potential ADHD-related probiotics in the gut, such as *Lachnospiraceae*, and boosts the production of SCFAs (79, 84). This increases the number of related metabolites and neurotransmitters, which affect the brain-gut axis and thus play a beneficial role in ADHD. Additionally, higher intakes of vitamin C can enhance collagen synthesis within the intestinal tract, which strengthens the intestinal barrier and indirectly impacts gastrointestinal health and the microbiome (79, 80). Furthermore, vitamin C is known to have a protective effect against inflammation, oxidative stress, and certain neurochemical factors and neurotransmitters, which are implicated in the pathophysiology of ADHD (85, 86). This evidence suggests that by modulating the gut microbiota, vitamin C may exert a positive influence on ADHD, a condition often associated with oxidative stress and neuroinflammations (81–83, 87, 88).

### 5.3 Beneficial effects of omega-3 polyunsaturated fatty acids on gut microbiota

Chili peppers also contain Polyunsaturated Fatty Acids (PUFAs) (87). These PUFAs are in the whole fruit and seeds, but their seeds show even greater concentration per gram (88). The main types of PUFAs include omega-3 PUFAs and omega-6 PUFAs, which are considered very important for human health. Unsaturated fatty acids cannot be produced by the human body and must be obtained from food (86). It has been reported that Omega-3 PUFAs deficiency exists in people with various mental disorders, including ADHD, depression, and Autism Spectrum Disorder (ASD) (89). A randomized controlled trial found that supplementing with omega-3 PUFAs mixtures increased the concentrations of EPA and DHA in red blood cell membranes and improved memory function in children with ADHD (90). In male animal models with ADHD, a diet rich in omega-3 PUFAs promotes increased dopamine (DA) and 5-HT turnover in the striatum, which enhances attention and reduces impulsivity (91). Children afflicted with ADHD present significantly decreased total omega-3 PUFAs in contrast to the control group, and the omega-6:omega-3 fatty acid ratio is conspicuously higher in children with ADHD compared to the control group (92). Therefore, correcting the omega-3: omega-6 imbalance can improve ADHD symptoms by stabilizing neuronal membranes, dopaminergic neurotransmission, and reducing neuroinflammation.

The earlier conversation highlighted how the gut microbiota influences ADHD. Growing research suggests that omega-3 PUFAs may influence the gut microbiota and its byproducts, contributing to the improvement of gut dysbiosis (93–96). Omega-3 PUFAs can directly affect the gut microbiota, while the gut microbiota can directly or indirectly regulate the absorption, bioavailability, and bioconversion of omega-3 PUFAs (97). Omega-3 PUFAs derived from food consumption, undergo partial metabolism by anaerobic bacteria like *Bifidobacteria* and *Lactobacilli* in the distal intestine, consequently affecting the composition of the gut microbiota (98). Studies involving randomized trials on omega-3 PUFAs supplementation in humans have demonstrated that such supplementation leads to a temporary rise in the populations of various bacteria responsible for producing SCFAs (99). There have been demonstrated that the addition of omega-3 PUFAs to the diet can raise the abundance and percentage of *Bifidobacterium* in the intestines of male Sprague–Dawley rats (97). A study evaluated the cognitive ability and social behavior of mice fed high and low doses of omega-3 fatty acids (DHA + EPA), and their microbial composition was also sequenced using 16S rDNA. The results showed that those who ate a diet rich in omega-3 fatty acids showed stronger cognitive function and had higher levels of lactic acid bacteria and bifidobacteria (100).

**Figure 1:**
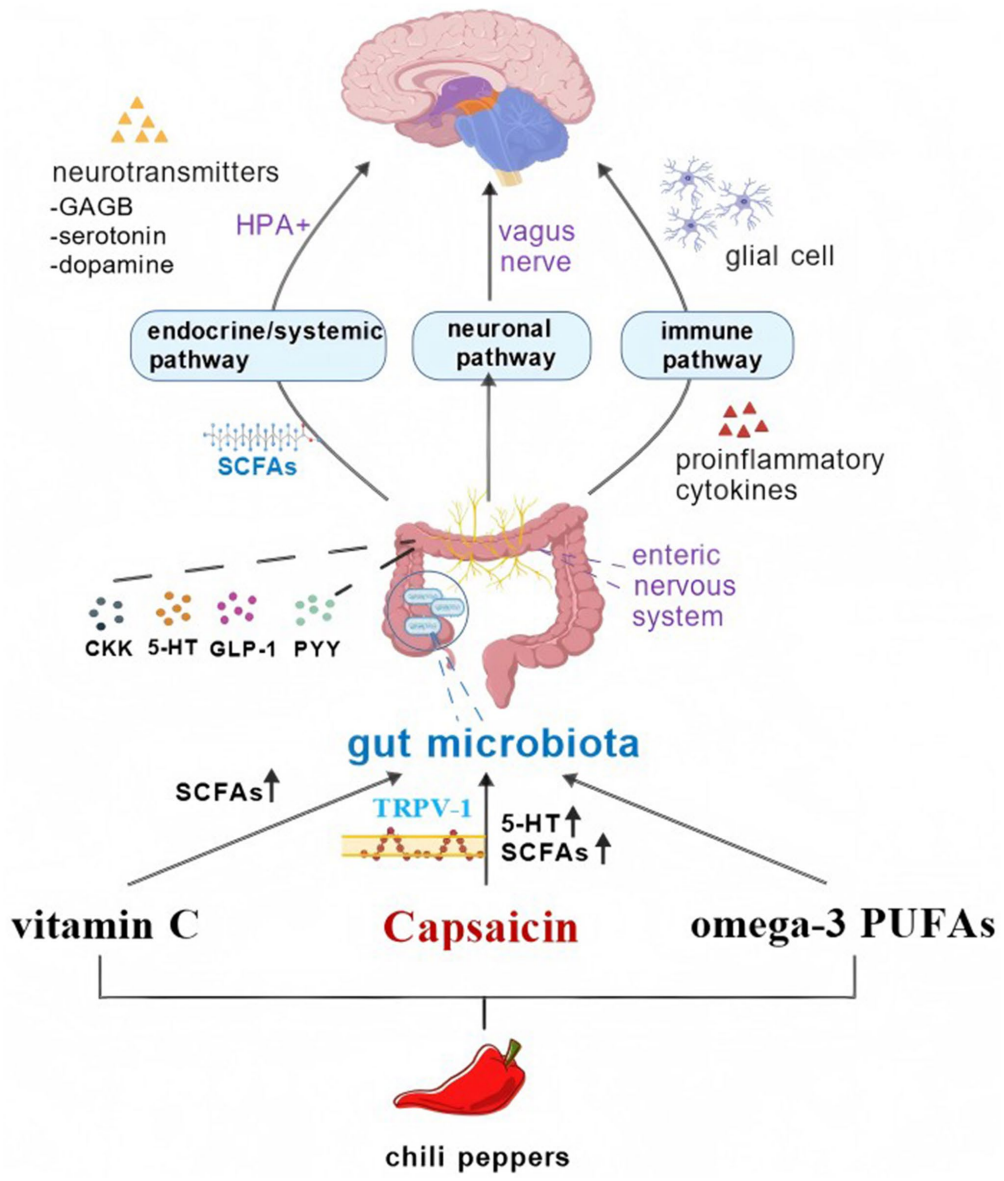
The active ingredient of chili peppers can affect the gut microbiota, thereby altering ADHD through the brain-gut axis. Figure created with BioGDP.com, licensed under CC BY-NC 4.0 (127).

Although there have been no clinical reports on the use of chili peppers in the treatment of ADHD, its active compounds (CAP, vitamin C, omega-3 PUFAs) have been shown to affect the gut microbiota and its metabolites (see Table 1). This interaction can provide a protective effect on the intestinal barrier, regulate the immune response and the production of neurotransmitters. It also provides anti-inflammatory and antioxidant properties. Peppers are a safe and relatively cheap compound that is commonly consumed in various countries. We suggest that regular addition of peppers to meals, such as bell peppers that can be eaten directly, may help improve symptoms in children with ADHD (Figure 1).

**Table 1:** To summarize the effect of active ingredients in peppers on gut microbiota.

Ingredient	Research object	Dose	Duration	Result	References
CAP	The MKN45-xenograft mice	50 mg/kg, 100 mg/kg	68 days	The ratio of *Firmicutes/Bacteroidetes* bacteria and the number of *Clostridium* were significantly increased	Deng et al. (64)
CAP	Four-week-old male C57BL/6 J mice	0.005% capsaicin/days	4 months	Dietary CAP can regulate the structure and number of gut microbiota and play a major role in the prevention of depression	Xia et al. (14)
CAP	*Vitro* model of the human gut microbiota	A total of 25 mL (concentration was 200 mg/L) of this solution once a day for day 1 and 7.5 mL twice a day from day 2 to 14 were injected into two bioreactors	14 days	Beneficial bacteria such as *Faecalis* and *Escherichia* have increased, the abundance of short-chain fatty acids increased	Mahalak et al. (5)
CAP	Eight-week-old female C57BL/6 J WT and B6.129×1-Trpv1tm1Jul/J (TRPV1^−/−^; KO) mice	2 mg/kg, once every other day	12 weeks	It enhanced the abundance of important intestinal microbial genera (such as *Bacteroidetes*, *Akkermansia*, and *Prevotella*), the abundance of short-chain fatty acids increased	Wang et al. (65)
CAP	Male Swiss albino mice (5–6 week old; 25 ± 3 g)	2 mg/kg	3 months	In the high-fat diet + capsaicin group, the abundance of *Ackermannia*, *Bacteriales*, *Pasteulosis*, and *Lactobacillus* was significantly increased. Capsaicin intervention significantly enhanced *A. muciniphila* abundance in the caecal contents	Baboota et al. (66)
CAP	Sprague–Dawley (SD) male rats	0.5 mg%, 1.2 mg%, and 1.7 mg%	8 weeks	Kochujang (1.2 mg%) intake decreased *Clostridium* and *Solibaculum* and increased *Blautia* and *Lachnoclostridium* belonging to the family *Lachinospraceae* compared to the Control group	Ryu et al. (63)
CAP	SD rats	2 g/kg (Gochujang，main bioactive compounds is capsaicin)	14 days	It is significantly increasing the number of *Akkermansia muciniphila* while decreasing the numbers of *Enterococcus faecalis* and *Staphylococcus sciuri*	Mahoro et al. (67)
Vitamin C	Healthy participants	1,000 mg/day	2 weeks	Daily supplementation of high-dose vitamin C led to an increase in the relative abundances of *Lachnospiraceae*, whereas decreases were observed for *Bacteroidetes*, *Enterococci* and *Gemmiger formicilis*	Otten et al. (78)
Vitamin C	Collagen-induced arthritis (CIA) mouse models	100 mg/kg	6 weeks	The results showed that treatment of CIA mice with vitamin C effectively rescued the gut microbiota imbalance and suppressed the inflammatory response associated with RA, and effectively alleviated arthritis symptoms in mice in which levels of the pro-inflammatory cytokines IL-6 and TNF-α were specifically reduced	Zhang et al. (82)
Vitamin C	Spontaneously hypertensive rat (SHR) model	200 mg/kg, 1,000 mg/kg	4 weeks	After being treated with vitamin C, the composition of the gut microbiota changed, showing greater diversity and abundance, close to the microbiota characteristics of rats with normal blood pressure	Li et al. (83)
Omega-3 PUFA	Healthy volunteers (*n* = 22)	2,000 mg EPA and 2,000 mg DHA	8 weeks	Omega-3 PUFA supplementation induces a reversible increase in several short-chain fatty acidproducing bacteria, independently of the method of administration	Watson et al. (99)
Omega-3 PUFA	SHR model	N-3 PUFA (EPA and DHA)-enriched feed (*n*−6/*n*−3 of 1:2.7)	postnatal days (PND) 25–50; PND 55–60	Dietary n-3 PUFAs may partly ameliorate ADHD-like behavior by reinforcement-induced mechanisms in males and partly via reinforcement-insensitive mechanisms in both sexes	Dervola et al. (91)
Omega-3 PUFA	SD rats	2,400 mg/day	12 weeks	Supplementation of ω-3 fatty acids could alleviate the inflammation of acne vulgaris by increasing the abundance of butyric acid producing bacteria	Huang et al. (96)

### 5.4 Chili peppers regulate gut microbiota to improve ADHD pathway

The exact mechanism of ADHD onset is still not fully understood. At present, it is clearly proposed that the abnormal reduction of DA level and the involvement and dysfunction of the corresponding brain region triggered by nervous system inflammation may be the main cause of ADHD (101). Previous studies have found that the abundance of bacteria associated with the production of neurotransmitters (such as GABA and DA) and pathogenic and opportunistic bacteria (such as *Enterococcus, Fusobacterium,* and *Eggerthella*) increased in the gut microbiota of children with ADHD, while the number of bacteria that play an anti-inflammatory role (such as *Bacillus faecalis*) decreased (101). Therefore, improving the gut microbiota or supplementation with probiotics is expected to alleviate ADHD symptoms by increasing the production of neurotransmitters in the gut, reducing intestinal inflammation, and reducing the abundance of intestinal pathogens. As mentioned above, various components of chili can act on the gut microbiota, so ADHD patients can benefit from it. The gut microbiota was commonly believed to control neurodevelopment through three pathways: the immune pathway, the neuronal pathway, and the endocrine/systemic pathway, with connections and interactions among them.

#### 5.4.1 Neural pathways

There are two neuroanatomical pathways that enable communication between the gut and the brain. First, direct interactions take place via the Vagus Nerve (VNS) and the autonomic nervous system (ANS) located in the spinal cord. VNS, which is the ninth cranial nerve, innervates the digestive tract by the hepatic and celiac branches. Siopi et al. (102) found that healthy mice receiving gut microbiota from chronic unpredictable mild stress (CUMS) mice showed vagus-mediated alterations in their 5-HT and DA pathways, which are associated with concurrent and persistent deficits in hippocampal neurogenesis and neuroinflammation. This suggests that the VNS can indeed mediate the effects of gut microbiota on brain function and behavior. In animal models, infections with pathogens such as *Campylobacter jejuni* and *Citrobacter Amalonac* induce anxiety-like behaviors (103), and supplementation with probiotics including *Lactobacillus rhamnosus* and *Bifidobacterium longum* alleviates these anxious/depression-like behaviors (104, 105). The gut microbiota has the ability to regulate host emotional and behavioral responses by acting on the vagus afferent nerve. Second, the enteric nervous system (ENS) of the gastrointestinal tract promotes bilateral interactions among these systems alongside ANS and VNS activity (106). The ENS, consisting of the myenteric and submucosal plexuses, communicates with the CNS via afferent neurons to transmit sensory information. This information travels through the spinal cord and vagal pathways, coordinating intestinal functions such as peristalsis and fluid flow (24). gut microbiota can also affect the maturation and function of ENS by activating pattern recognition receptors (PRRs), such as Toll-like receptors (TLRs) and 5-HT receptors (107).

In addition, the gut microbiota may influence the production of neurotransmitters. For example, certain *Clostridium perfringens* regulate the synthesis of 5-HT by expressing tryptophan hydroxylase 1 (TPH1) (108). The gut microbiota also influences DA levels in the frontal cortex and striatum of rodents, and perturbations of the dopaminergic system are associated with mood-related disorders and cognitive dysfunction. Besides, gut microbiota can regulate the expression of brain-derived neurotrophic factor (BDNF), which is closely related to neurogenesis, thereby affecting cognition and attention (109).

#### 5.4.2 Endocrine/systemic pathways

Gut microbiota play a crucial role in the development and function of the HPA axis, a key regulatory system for neuroendocrine signaling and stress responses (110). The gut microbiota is capable of generating diverse metabolites, releasing neurotransmitters and neuromodulators, thereby influencing the HPA axis and exerting an effect in the pathogenesis of ADHD. When germ-free mice were exposed to constraint stress, they exhibited a stronger stress response compared to normal mice, with significant increases in adrenocorticoid and glucocorticoid levels. Adding *Bifidobacterium* can reduce the intensity of stress response in germ-free mice, suggesting that the gut microbiota plays a role in regulating the HPA axis (111). Studies have shown that the gut microbiota not only directly affects the production of glucocorticoids and immune mediators, such as tumor necrosis factor-α (TNF-α), interleukin-1β (IL-1β), and IL-6, but also further stimulates HPA axis activity through these mediators (112). The substances or metabolites mentioned encompass *γ*-aminobutyric acid (GABA), 5-HT, DA, norepinephrine, acetylcholine, histamine, SCFAs, and others (113, 114). It has been confirmed that *bacilli* and *Bifidobacteria* can produce GABA, *Escherichia coli*, *Bacillus*, and *yeast* can produce norepinephrine, *Candida albicans*, *Streptococcus*, *Escherichia coli*, and *Enterococci* can produce 5-HT, and *Bacillus* can also produce DA (115). However, metabolites such as 5-HT are unable to cross the blood–brain barrier without the involvement of VNS. VNS acts as a significant link between the endocrine/systemic pathway and the neuronal pathway. Furthermore, the gut microbiota interacts with the enteric endocrine system to regulate neurodevelopment. Enteric endocrine cells can produce glucagon-like peptide-1 (GLP-1), peptide YY (PYY), cholecystokinin (CCK), substance P, and 5-HT, which are involved in metabolic functions related to energy homeostasis (116). At the same time, enteric endocrine cells can sense various microbial signals and can also connect with VNS to communicate with the CNS. Gut microbiota is also capable of producing SCFAs, particularly butyrate, propionate, and acetate, which combat oxidative stress in inflammatory states by upregulating the activity of antioxidant enzymes such as glutathione peroxidase (GPX) and catalase (117).

#### 5.4.3 Immune pathways

The gut microbiota plays a key role in regulating the differentiation and maturation of innate immune cells (such as macrophages, innate lymphocytes and dendritic cells), and is essential for the formation and integrity of the intestinal barrier and blood–brain barrier. Studies have shown that changes in the gut microbiota down-regulate the expression of tight junction (TJs) proteins, leading to impaired intestinal barrier and blood–brain barrier function, allowing biomacromolecules and microorganisms to penetrate the barrier, which in turn triggers neuroinflammatory responses (118). In addition, the gut microbiota was found to influence the process of monocyte migration from the periphery to the brain mediated by TNF-α, which was reversed by the use of probiotics (119). The development and maturation of the acquired immune system is also dependent on the presence of the gut microbiota. For example, intraepithelial lymphocytes require *L. reuteri* as well as a tryptophan-rich diet to reprogram intraepithelial CD4^+^T cells into immunomodulatory T cells (120). At the same time, intestinal microbiota dysbiosis leads to decreased levels of immunoglobulin A and G1 (IgA and IgG1) and increased levels of immunoglobulin E (IgE), thereby increasing the risk of developing a variety of diseases (121). SCFAs also have immune functions. They can reduce damage to the intestinal epithelial tissue, regulate tight junctions, and enhance the intestinal immune barrier. Other functions of SCFAs include regulating the production of cytokines by bone marrow cells, as well as differentiating T regulatory cells and promoting the differentiation of helper T cells (122). In addition to regulating the intestinal barrier, the gut microbiota is also involved in the transport of immune cells from the intestine to the brain. For example, the gut microbiota activates a group of IFNγ ^+^ NK cells, which then migrate to the CNS and induce the production of anti-inflammatory astrocytes.

It can be seen from this that gut microbiota can regulate brain immune responses and cells in this way, and changes in the composition of gut microbiota can lead to dysregulation of the MGBA, ultimately leading to ADHD. Chili peppers are an indispensable part of dietary structure, which contain CAP, vitamin C, and fatty acids that can alter the composition and function of the gut microbiota and affect the generation of fatty acids. These changes are transmitted through the brain-gut axis and subsequently affect ADHD.

## 6 Conclusion

Due to the long-term negative effects and side effects of medications and behavioral treatments, the search for effective treatments for ADHD has been ongoing in recent years. The gut microbiota maintains an interactive relationship with the host through neural, endocrine, and immune pathways, which is known as MGBA. Changes in MGBA are a factor in the development of neurodevelopmental disorders in children. Children with ADHD exhibit neuroinflammation and oxidative stress, which are considered part of its pathophysiological mechanisms. The MGBA may play a certain role in this process. According to MGBA theory, the effect of diet on the gut microbiota may be a promising therapeutic approach for children with ADHD. Peppers, as a common and inexpensive spice, are widely used around the world. Peppers not only are a unique flavor to food but also are rich in various beneficial components, such as CAP, vitamin C, and fatty acids. They can alter the composition of the gut microbiota and the secretion of metabolites, exert anti-inflammatory and immunomodulatory effects, and thus potentially have a positive impact on ADHD through the MGBA. Using small amounts of chili peppers in the diet as an adjunct to treating ADHD has fewer side effects and is more acceptable to parents compared to traditional medication. This review describes the effects of gut microbiota and dietary factors on ADHD, while exploring the potential therapeutic advantages of chili peppers in modulating gut microbiota through MGBA in children with ADHD. This potential mechanism and these effects are derived based on the aforementioned research and related theories; however, at present, there is still a lack of large-scale clinical research evidence to fully support this. This conclusion helps us further clarify future research directions, such as large-scale, randomized, double-blind, placebo-controlled human clinical trials, to validate the benefits and risks of chili peppers and CAP consumption for neurological disorders, especially ADHD.

CAP is widely used around the world, but the debate over its safety continues. A large number of studies have confirmed that CAP has anti-tumor, anti-inflammatory and analgesic effects (123). Contrary opinions suggest that long-term use of high doses of CAP may increase the risk of developing tumors (124, 125). Significantly increased consumption of chili peppers or CAP has the potential to increase the risk of gastric cancer, however, inconsistencies still exist in subgroup analysis between different regions (126). On the contrary, one study suggests that repeated ingestion of CAP over a prolonged period reduces symptoms in functional dyspepsia (128). The long-term safety of regular chili consumption has not yet been established, and further research is needed on the safety and potential chronic effects of long-term chili and CAP intake. Exploring the minimum effective dose required to achieve therapeutic effect and minimizing potential side effects is necessary to enhance the clinical effectiveness of capsaicin-based therapies. Chili peppers can also irritate human skin, and excessive intake can cause nausea, vomiting, abdominal pain and burning, diarrhea. Contact of CAP with eyes can cause severe tearing, pain, conjunctivitis, eyelid spasms. But combining CAP with other phytochemicals can reduce these adverse effects, such as vanillin, flavonoids, alkaloids, cannabinoids (129–131). However, there may be individual differences in tolerance to chili peppers, depending on existing health status or genetic susceptibility, etc. In the future, we will explore the factors that influence an individual’s response to chili peppers and CAP to develop personalized dietary guidelines.

There’s no doubt that the health effects of chili peppers are mostly positive. CAP, in particular, has potential benefits in treating ADHD. However, most of the available evidence comes from animal or *in vitro* studies, and these findings have not been consistently replicated in human clinical trials. When extrapolating the results of animal experiments to humans, the differences between animals and humans should be considered. In the future, more experimental studies and high-quality large-sample clinical trials are still needed to explore the effects of spicy components on mental disorder diseases through the microbiota-brain-gut axis. Most of the studies had short intervention periods, which does not shed light on the long-term effects of chili consumption on ADHD symptoms. The composition of gut microbiota is affected by race, region, diet, and other factors, and there are direct and significant differences among different individuals. Future studies need to analyze the characteristics of gut microbiota in different populations in more detail, and combine multi-dimensional data (such as genomics, metabolomics, etc.) to reveal the specific mechanisms of gut microbiota in mental disorders. Furthermore, for patients of different ages and races, the appropriate intake of chili peppers needs to be tested in future studies to explore the minimum effective dose required to achieve therapeutic effects and minimize potential side effects. In addition, the above-mentioned pungent components have the characteristics of low bioavailability and high liver and intestinal circulation intervals; therefore, looking for more efficient derivatives or complexes and choosing convenient and fast administration routes will bring new breakthrough drugs.

In the later stage, we will further improve the clinical and basic research to clarify the impact and mechanism of peppers on ADHD. Future studies should cover populations of different ages, genders, and regions to fully assess the range of safe doses of chili peppers in different populations and their ongoing effects on ADHD symptoms. This will provide a scientific basis for personalized treatment and promote the clinical application of Capsicum as an adjunct therapy. We should also consider that different types of chili peppers may produce different effects due to their intensity and active ingredient content. We also need to validate pharmacokinetic/safety studies of CAP in patients with psychiatric disorders such as ADHD. We can also improve the pharmacological properties of the compound by modifying the chemical structure of CAP, overcome some adverse reactions and increase the acceptability of peppers.
